# Child feeding practices in a rural Western Kenya community

**DOI:** 10.4102/phcfm.v1i1.15

**Published:** 2009-05-07

**Authors:** Grace M. Mbagaya

**Affiliations:** 1 Department of Family and Consumer Sciences, Moi University, Kenya

**Keywords:** Kenya, feeding, infants, rural, exclusive breastfeeding

## Abstract

**Background:**

Breastfeeding is nearly universal in Kenya. However, supplementation of breast milk starts too early, thereby exposing the infants to diarrhoea and other infections. Despite the recom-mendation of the World Health Organization (WHO) of exclusive breastfeeding (EB) from birth to six months, EB is rare and poorly timed and complementary feeding (CF) practices are still common. The study describes feeding practices of children aged 0 to 24 months in the Mumias Division of the Kakamega district in Kenya.

**Method:**

Using a cross-sectional study, 180 mothers of infants/children were interviewed using a structured questionnaire. Data on socio-demographic characteristics, feeding practices and sources of information on the same were obtained from the mothers.

**Results:**

Whereas 92.1% of the children were breastfed, only 12.2% of the mothers practiced EB up to 4 to 6 months. Mothers introduced liquids and complementary foods at a mean age of 2.7 months and by the fourth month, more than one-third (34.5%) of the mothers had initiated CF. Apart from water, fresh milk, tea, commercial juices, maize-meal/millet porridge, mashed potatoes, bananas and fruits were also introduced. The perceived reasons for introducing these foods included the child being old enough (33.8%), another pregnancy (25%), insufficient milk (20.3%), sickness of the mother or child (10.5%) and in order for the child to eat other foods (11.4%). Over half (53.3%) of the mothers obtained information on BF and CF from friends, neighbours, media advertisements and health workers.

**Conclusion:**

Breastfeeding is common; however, mothers do not seem to practice the WHO recom-mendations. Mothers in this study area and other rural communities need to be empowered with information on the correct BF and CF practices through existing government health services, non-governmental organisations and other community-based networks, especially in the light of the HIV/AIDS pandemic.

## INTRODUCTION

The biological benefits of breast milk and breastfeeding (BF) for mothers and infants in both developing and industrialised countries are well documented.^1^ Recent research findings have demonstrated physiological, immunological, psychological and economic factors in favour of BF for up to six months of life. ^2–6^

BF and complementary feeding (CF) involve a combination of practices to maintain breast milk intake and at the same time ensure that the correct quality and quantity of foods are consumed by children.^7–9^ Early cessation of BF and poorly timed CF are still common, especially in developing countries such as Kenya. The World Health Organization (WHO) and other United Nations agencies recommend that children should be breastfed, with the introduction of safe and nutritionally adequate foods from about 4 to 6 months, until they are at least two years old.^10–12^ BF and CF behaviours are important predictors of infant and child nutrition, health and survival and thereby contribute to the well-being of the future generation. Improving CF practices includes the timing of introduction of complimentary foods and the types and amounts of foods introduced.^13, 14^ The appropriate age of introducing complementary foods has been a topic of much discussion.^15–17^ Current recommendations and practices for BF initiation and CF practices reveal some similarities as well as some differences among mothers in developing countries.^18^

Studies indicate that the premature introduction of CF may lead to earlier cessation of BF,^19, 20^ and early CF of infants has been associated with a shorter duration of postpartum infertility.^21–23^ In Lima, Peru, rural Indonesia and Western Kenya, the introduction of complementary foods was associated with earlier termination of BF even when controlling for nursing frequency.^24, 25^ A study in northern Thailand suggests that foods given by bottle have more adverse impacts than foods given by other means.^26^ Similarly, findings from a study in Honduras revealed that the early use of formula was related to early cessation of BF, whereas other foods had no impact.^27^

It has been observed that complementary diets offered to many infants in sub-Saharan Africa are monotonous, bulky and rarely cover the shortfall left by breast milk in providing the energy and nutrients required to support rapid growth, build nutrient stores and assure resistance to infections.^28^ For the majority of children in developing countries, growth stunting occurs from several months of birth to about two years of age.^29^ This coincides with the time when solid and semi-solid foods are introduced into the diets of infants.^30–32^ Continued BF into the second year of life has been found to have positive effects on the growth of the child.^33, 34^

This study aimed at establishing to what extend WHO infant-feeding recommendations are being adhered to in this rural Kenyan community. BF and CF patterns of mothers of infants/children were investigated from birth to 24 months. The study considered the timing of CF, the foods that are usually introduced, perceived reasons for introducing other foods and the sources of information on BF and CF.

## METHOD

This was a cross-sectional study carried out between November 1998 and December 1999 in 12 villages of the Isongo sub-location, East Wanga (Shibinga) location, Mumias division, Kakamega district, Kenya. The 12 villages are geographically, socio-economically and culturally similar. The division is well served with schools and market places. The study area (Isongo sub-location) has seven primary schools, two secondary schools and one government health centre (Makunga), which serve the Mumias and Lurambi divisions. There is one mission hospital and a few private hospitals and clinics. Most of the residents in the Isongo sub-location are within four to five kilometres away from the Makunga health centre. Electricity remains a dream for most of the residents in the area, although there is a major sugar company in the division that has power.

A random sample of 180 children aged 0 to 24 months was selected from 566 preschool children using a formula suggested by Fisher.^28^ The formula was found to be appropriate for this kind of household survey intended to cover an area such as this. The method ensured that all eligible children from the sampled sub-location had an equal chance of being included in the survey. All 12 villages of the Isongo sub-location were covered in the study. Each household was visited once and the mother of the child interviewed. Information on demographic, socio-economic and child characteristics, which included BF and CF practices, was collected. The information on feeding practices was ascertained by recall information from the mothers.

The questionnaires, which were in English, were translated into Kiswahili (the national language) and then back-translated into English. The interviews were conducted in Kiswahili. Pretesting and practical interviewing exercises were conducted repeatedly among the research assistants and mothers from the neighbouring location before carrying out the actual survey. Semi-structured interviews were conducted with key informants, who included three nurses/midwives, one clinical officer, one nutrition field worker and two community workers, who were randomly sampled from staff at the Makunga health centre. Findings from these interviews have been incorporated in the relevant sections of the study.

The completed questionnaires were checked for completeness and accuracy on a daily basis by the principal investigator. The data were entered into computer using the Dbase IV programme for Windows 95. The Dbase IV files were imported and converted to SPSS Version 7.0 for Windows 95 for cleaning and statistical analysis. Descriptive statistics were used in data analysis. Responses from some of the qualitative data were coded and frequencies determined. Cross-tabulations were used in establishing relationships between variables at a significant level (p < 0.05).

## RESULTS

### Demographic characteristics

The sample comprised of 180 mother-infant/child pairs. A total of 51.2% of the children were males, while 48.8% were females, with a mean age of 13.5 months. Over half (66.1%) of the children were between 1 and 12 months. The rest (33.9%) were between 13 and 24 months. Mothers were the main caregivers of children in this community. However, immediate older siblings, grandmothers, maids or household helps also assisted with the care of the children. In a few (36.1%) of the households, mothers took care of the children without any assistance. Due to this support care, mothers were able to attend to other household chores. A total of 11.1% of the mothers had no formal education, 61.2% had primary education, while 27.7% had secondary education. The mothers’ ages ranged between 15 and 50 years with the majority (50%) aged between 20 and 34 years.

### Breastfeeding and introduction of water

From recall information, 95.2% of the mothers indicated having initiated BF immediately after birth. The study's findings indicate that exclusive breastfeeding (EB) up to 4 to 6 months was only practiced by 12.2% of the mothers. A mean duration of EB of 2.9 months was reported. More than half (51.5%) of the mothers gave plain water as the first additional fluid to their children when most of the infants were between 1 and 2 months. Mothers gave a number of reasons for giving water to the babies, which included that breast milk is food, but water is needed as a fluid for better digestion, breast milk contains water but it is too sweet and fatty therefore extra water is necessary, infants become thirsty just like adults therefore they need water and water makes the baby feel full for longer.

### Complementary feeding

Mothers were asked at what age they introduced breast milk substitutes or complementary foods to their children apart from breast milk and water. Recall information on CF processes showed that EB was short-lived. Table 1 shows the ages of initiation of complementary foods. The mean age of introducing complementary foods was 2.7 months. In addition to plain water, 40% of the mothers introduced milk to which water had been added, while fresh and commercially prepared juices and tea were given to the infants at about two months. This would be followed with porridge (a thin gruel) made from maize/ sorghum and millet flours. A few (24.3%) of the mothers enriched this porridge with milk, eggs, orange or lemon juice, groundnut paste, margarine and other ingredients. A small number (9.4%) of mothers gave commercial cereal products such as Cerelac. Mashed green bananas and in rare cases Irish potatoes and other semi-solid foods would be given between the third and fourth months. 16% of the mothers indicated giving fruits such as mangoes, papaws and sweet bananas to the infants/children. Half (50%) of the mothers indicated giving *ugali* (a national dish of stiff porridge usually served with accompaniments) and some gruel to the children at six months.

**TABLE 1:** Age of initiation of complementary foods

AGE IN MONTHS	NO. OF CHILDREN (N = 180)	%
**≤** 4	62	34.5
5–7	53	29.4
8–11	36	20.0
12–14	27	15.0
≥ 15	2	1.1

Overall, by the seventh month, more than half of the infants (63.9%) had been introduced to complementary foods, as shown in Table 1. Twenty per cent of the mothers introduced complementary foods between the eighth and eleventh months. Only two (1.1%) mothers indicated having introduced other foods when their children were older than 15 months. These mothers reported having had enough milk to breastfeed.

### Perceived reasons for stopping breastfeeding and introducing new foods

The mean duration of BF was 14.5 months and 45.3% of the mothers stopped BF when the children were 18 months old. Mothers advanced a number of reasons for introducing other foods. These included the child being old enough, another pregnancy, the child refusing breast milk, insufficient milk, the mother or child being sick and in order for the child to eat other foods, as indicated in Table 2. The mothers’ ages and educational levels had no influence on the BF and CF patterns. Over half (53.3%) of the mothers obtained information on BF and CF from friends/neighbours while (24.1%) from media advertisements, 15.5% from the health clinics and (6.1%) from other sources.

**TABLE 2:** Reasons for stopping breastfeeding

REASONS	NO. OF MOTHERS (N = 180)	%
Child old enough	61	33.8
Another pregnancy	45	25.0
Insufficient milk production	25	13.9
Child refused	20	11.2
Mother/child sick	15	8.3
To eat other foods	14	7.8

**TOTAL**	**180**	**100**

## DISCUSSION

Breastfeeding was considered a natural method of infant feeding in this rural community. Mothers did not need encouragement to breastfeed as they did it willingly. The fact that mothers in the study sample breastfed their children almost immediately after delivery is not surprising, as BF is reported to be a universal practice that is initiated soon after birth in many developing countries, particularly in sub-Saharan Africa.^9, 11, 15, 19, 21^

The present findings indicate that only 12.2% of the mothers practiced EB. This is comparable to 17.0% reported in the latest Kenya Demographic Health Survey.^8, 9, 29^ The mean duration of exclusive BF of 2.7% months observed in this study is slightly lower than the national mean of 2.9%.^12, 30, 31^ This could be due methodological and age categorisation differences.

The WHO recommendation of EB up to 4 to 6 months^8^ and revised to about six months of age with complementary foods thereafter^12^ are not widely practiced in Kenya. This is similar to other developing countries of the world, particularly those in sub-Saharan Africa.^1, 4, 7–9, 14, 15^ The proportion of infants under four months of age who are exclusively breastfed is highest (82%) in Asia followed by 63% in Nepal, North Africa (Morocco) and Latin America.

Findings from this study indicate that BF and water supplementation are predominant practices. This is comparable to findings from the country's National Nutrition Survey, in which sugar and water were fed to 26% of the infants from birth to about three months of age.^22, 26, 29–31^ Liquids and complementary foods are introduced at a fairly young age for this typical rural community. This has been attributed to mothers having to stop BF mainly due to new pregnancies, a perceived lack of sufficient milk, beliefs that breast milk is not adequate and mothers wanting to introduce other foods. The practice of introducing prelacteal feeds at a young age seems to be similar to observations made elsewhere.^17^ For example, in the Indian subcontinent and parts of south-east Asia there is a strong belief that colostrum is highly undesirable and prelacteal feeds of sweetened water, goats milk and diluted cow milk are normally given to infants during the first three days after delivery.^9, 13, 14, 23, 24, 32^

New pregnancies were cited as one of the reasons for stopping BF. In a number of studies, BF during a new pregnancy is usually discouraged as it is considered harmful to the mother, foetus and baby.^2, 3, 22, 25, 26, 28, 32^ In this study, 25% of the mothers stopped breastfeeding as soon as they realised they were expectant. Findings from a children's study in the same locality indicate that new pregnancies precipitated the termination of BF.^25, 26^ The findings are consistent with the findings of a study in Uzbekistan where BF during pregnancies is discouraged.^15^ In behavioural studies in the developing countries, pregnancy is frequently cited as a reason for weaning^15, 16, 18, 20^ and the belief that pregnancy and lactation are incompatible states is widespread.^5, 16, 22^ Breast milk insufficiency was also reported as a reason for stopping BF. The perception of insufficiency is often based on the crying of the infant. This is comparable to a number of studies in Kenya and elsewhere. ^1, 2, 5, 11, 33^

Beliefs regarding CF have implications for child nutrition, since the age at which children are reported to be most vulnerable to growth faltering is the period between six and eighteen months, which is the period of transition between breast milk and an adult diet.^2, 15, 17^ The transition period varies within cultures, for example in Bangladesh the period between 13 and 18 months is considered most crucial in a child's development.^32–34^ The appropriate time of initiation of CF also varies across cultures with the earliest incidence of CF seen in Indonesia,^24, 32–34^ where rice and mashed bananas are introduced in the first week of life. This belief is supported by the assumption that children who are fed a meal will be calmer and sleep more and this will help the mother carry on with her work. In Egypt it is believed that supper after 40 days of full BF is necessary for promotion of growth and fatness.^17^ Water, cow milk, maize and millet porridge were popular supplements in this study. The findings are similar to what has been reported in Kenya's National Nutrition Survey.^12, 29, 31^

There were no significant interactions between BF and CF patterns with the age and educational levels of the mothers. This is not a surprising trend, as similar patterns have been reported elsewhere in the developing countries.^16, 17, 20, 22–24, 34^ In general, mothers believe that babies need breast milk, however, it is believed that water, fruit juices and other semi-solid foods are needed before six months of age. The mothers believe that, after six months, babies still need breast milk but that they should be gradually trained to enjoy the family diet by being given juice, milk, tea, mashed or boiled vegetables, bananas, Irish potatoes, porridge and *ugali*. By the time teeth appear, they should begin eating ordinary family foods.

### Conclusion

From this study, it can be concluded that nearly all mothers in the Isongo sub-location successfully breastfed their babies from birth to at least 12 months and others for much longer. Some practices that make it difficult for mothers to promote appropriate complementary feeding from six months with continued BF to two years and beyond are highlighted in the study. There is definitely a lack of accurate information on the timing and the foods given to the infants/children during this period (0–24 months).^34^

There is a need for a more comprehensive and proactive policy on BF and CF in this area and many other similar areas in the country. The policy may be integrated into the different health services provided by government and voluntary and non-governmental organisations at community levels. Continued BF needs to be actively protected and encouraged due to its documented benefits. However, this should be done with caution, especially by HIV-infected mothers.

It is suggested that mothers be trained on BF and CF and be used as village nutrition educators in this area. Experience from other developing countries shows that such community participation is an essential component of sustainable development programmes.^15^

### Ethics approval

The Ministry of Research Science and Technology approved the research by granting a research permit. Contacts were established with the District Commissioner's office, the chiefs, assistant chiefs and the village headmen. The District Health Management Team approved the use of the health facility for information and discussion during the study. All the study procedures were explained to the mothers and their verbal consent sought before their involvement in the study. The use of numbers on the questionnaires instead of names ensured confidentiality. When appropriate, interviewers counselled mothers on infant/child feeding.
